# Clinical Reasoning and Challenges Faced With Onset Psychotic Symptoms in a Case of Combined Huntington’s Disease and Gayet-Wernicke Encephalopathy

**DOI:** 10.7759/cureus.61875

**Published:** 2024-06-07

**Authors:** Moujib Omri, Mohamed Ferhi, Mariza Oliveira Galvao, Oliver Hamm

**Affiliations:** 1 Department of Psychiatry and Psychotherapy, Klinikum Mutterhaus der Borromäerinnen, Trier, DEU; 2 Department of Psychiatry, Mohamed Taher Maamouri University Hospital, Nabeul, TUN

**Keywords:** new-onset psychosis, schizophrenia, alcohol dependence, organic delusional disorder, wernicke encephalopathy, huntington's disease

## Abstract

Physicians are occasionally confronted with patients presenting psychotic symptoms of organic origin. Therefore, precision in diagnosing the organic basis is pivotal for targeted treatment, addressing the underlying etiology. This case study delineates the nuanced phases of clinical reasoning employed to ascertain a diagnosis of Huntington's disease (HD), notably amidst concurrent alcohol dependence. A comprehensive clinical examination and meticulous review of the patient’s medical history served as linchpins in guiding subsequent investigations toward identifying the etiological underpinnings of the psychotic symptomatology. Furthermore, this case sheds light on the uncommon overlap of HD and Wernicke's encephalopathy, compounding diagnostic complexities, especially given the polymorphic nature of HD. The diagnostic intricacies needed precise analysis of the clinical picture and a deep understanding of potential interactions between neurological pathologies and the deleterious effects of alcoholism on the nervous system.

## Introduction

Huntington’s disease (HD), initially documented by George Huntington in the Medical and Surgical Reporter of Philadelphia in April 1872, is a rare hereditary disorder bearing his name [1]. While acknowledged as a prevalent degenerative neurological condition, its occurrence is estimated at 5-10 cases per 100,000 individuals in Europe [2]. The disease follows a Mendelian dominant inheritance pattern, stemming from an expansion of the CAG triplet within the huntingtin gene [3,4]. Clinically, its onset typically manifests with choreic motor symptoms and psycho-cognitive disturbances such as prodromal depression and dysexecutive syndrome, accompanying progressive degeneration of the striatum and other cerebral structures [3].

Although HD is frequently recognized for its neurological features, its infrequent presentation with psychotic symptoms can obscure early diagnosis, often mimicking primary psychiatric conditions [3,4]. Cognitive symptoms are a hallmark of HD, often emerging subtly years before clinical onset and progressing to pronounced subcortical and frontal dementia in advanced stages [4]. Additional cognitive deficits include executive dysfunction, general psychomotor slowing, and impaired short-term memory. Psychiatric symptoms are widespread, with depression affecting approximately 40% of HD patients, making it the most common psychiatric condition. Anxiety follows as the second most prevalent psychiatric issue. Other psychiatric manifestations include irritability, aggression, obsessive-compulsive behaviors, and, less frequently, psychosis [4]. Establishing a definitive diagnosis can be complex, especially in cases where substance use disorders, such as alcohol dependence, coexist. This article delves into a rare instance of HD, characterized predominantly by psychotic manifestations, in a patient grappling with concurrent alcohol dependence. The present case report aimed to delineate the diagnostic trajectory and the sequential steps that culminated in identifying HD, a process that was further complicated by the absence of overt motor neurological signs and the substantial influence of alcoholism.

## Case presentation

Diagnosis of schizophreniform disorder

This case study examines the complex medical history of a 29-year-old unmarried woman, residing alone in a socioeconomically disadvantaged environment. Raised by adoptive parents with no knowledge of her biological father, she underwent multiple hospitalizations during adolescence due to self-harm and impulsive behavior such as neglect of personal safety and risky decision-making. At the time, these behaviors were attributed to an impulsive trait devoid of a formal personality disorder diagnosis. By age 17, these behaviors had subsided. However, recent events necessitated her urgent admission to our psychiatric unit after neighbors alerted the police to her acute agitation and dangerous actions like attempting to set fire to her residence and damaging furniture. Upon arrival, authorities observed a decline in personal hygiene, with evidence of excessive alcohol consumption and inhalation of deodorant spray. Despite regular alcohol use since age 16, with occasional deodorant spray inhalation, she denied using other substances. A psychiatric evaluation revealed psychomotor agitation, irritability, incoherent speech, bizarre behavior, auditory hallucinations, and abulia, which persisted even after alcohol withdrawal. While physical and neurological exams, urine drug screens, and brain scans were unremarkable, the patient declined brain magnetic resonance imaging. Following over six weeks of symptoms, she received an initial diagnosis of schizophreniform disorder alongside alcohol dependence. The administration of lorazepam and olanzapine resulted in a reduction in agitation and hallucinations yet failed to significantly alleviate the negative symptoms.

Diagnosis of Wernicke's encephalopathy

**Figure 1:**
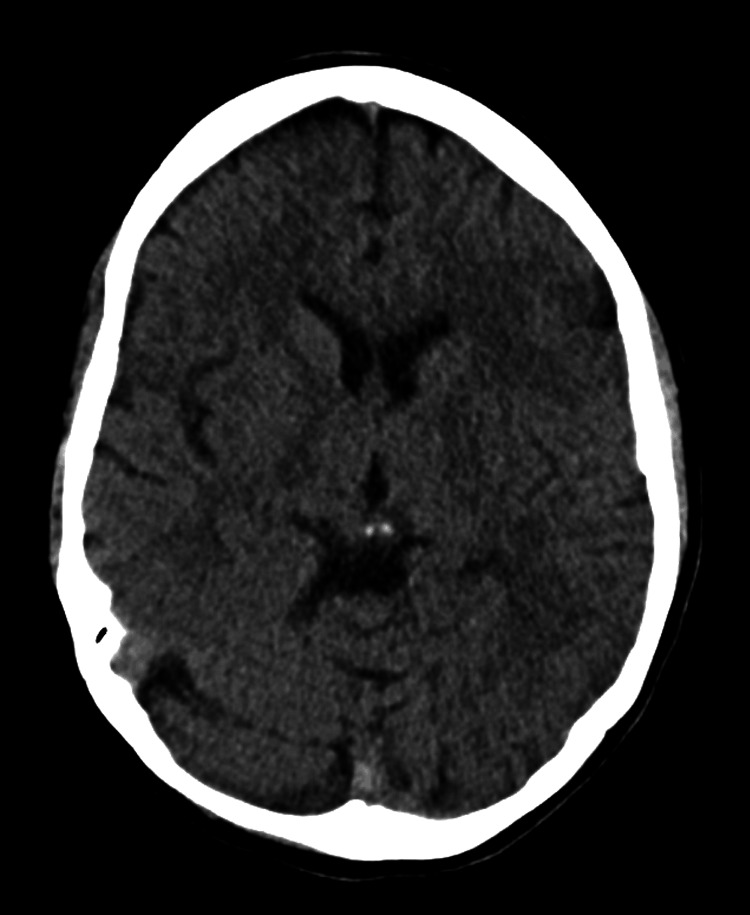
Computed tomography of the head suggesting a normal imaging study

Three months later, the patient returned to the emergency department, accompanied by law enforcement, exhibiting temporal disorientation and motor agitation. Clinical evaluation disclosed oculomotor abnormalities, including diplopia and nystagmus, along with mild hepatic cytolysis, cholestasis, and macrocytosis, which are indicative of chronic alcoholism. Following the exclusion of expanding intracranial mass via computed tomography (Figure 1), Wernicke's encephalopathy was suspected and promptly addressed with alcohol withdrawal and thiamine therapy. Subsequent resolution of symptoms, notwithstanding the absence of cerebral magnetic resonance imaging due to the patient's non-cooperation, corroborated the provisional diagnosis. Post-discharge, the patient received olanzapine and melperone, with an alcohol dependence monitoring liaison assigned to the patient.

Diagnosis of HD

A third hospitalization within four months ensued when the patient presented with acute psychotic symptoms, notably imperative and offensive acoustic hallucinations, accompanied by cognitive dysfunction and memory impairment. The patient denied substance use and claims to have been abstinent from alcohol since her last discharge. Her neurological examination was unremarkable, albeit the Mini-Mental State Examination (MMSE) revealed mild cognitive impairment with a score of 22/30. Further investigation unveiled a familial history suggestive of HD, prompting genetic testing confirming the diagnosis-excessive CAG repeats (47±1) on one of the two huntingtin alleles on chromosome 4. Despite the therapeutic efficacy of clozapine against hallucinations, memory deficits persisted. Psycho-educational sessions and a referral to an HD specialized center post-discharge were organized. Currently, the patient undergoes regular psychiatric outpatient follow-up, exhibiting progressive cognitive decline without motor symptoms to date.

## Discussion

The patient, a young woman, initially presented with an acute psychotic episode accompanied by behavioral disturbances, but without any apparent neurological signs. This led to a diagnosis of schizophreniform disorder after the exclusion of an organic etiology such as substance-induced psychotic disorder and cerebral tumors. She was treated with antipsychotic medication. Three months later, she was readmitted to the hospital with Wernicke's encephalopathy, which was characterized by oculomotor dysfunction and mental confusion. This was attributed to chronic alcohol intoxication. Emergency treatment, including alcohol withdrawal and thiamine administration, resulted in regression of neurological symptoms. After persistent cognitive deterioration, HD was suspected, supported by a family history, and confirmed by genetic testing revealing an increase in CAG repeats. Clozapine, administered to treat acoustic hallucinations, had a moderate effect without significantly improving the effects of cognitive deterioration. The patient continued to show cognitive deterioration without motor signs and is currently receiving outpatient psychiatric treatment with appropriate psychoeducational support.

In the existing literature, there is a paucity of evidence to suggest that a first psychiatric manifestation in the form of a “schizophrenia-like” psychotic disorder is a rare occurrence in HD [3,5]. However, some authors reported such cases. Table 1 provides a summary of five recent cases. To the best of our knowledge, no concurrent association between HD and Wernicke's encephalopathy has yet been reported.

**Table 1:** Recent cases of Huntington's disease revealed by psychosis

First Author	Year	Age	Sex	Country	Psychiatric Presentation	Number of CAG Repeats
Sengul et al. [6]	2014	28	Female	Turkey	Paranoid delusions and hallucinations	Not available
Nagel et al. [7]	2014	44	Female	Germany	Behavioral disorders and psychosis	Not available
Lobna et al. [8]	2018	32	Female	Tunisia	Paranoid delusions and behavioral disorders	Not available
Gonçalves et al. [9]	2021	32	Male	Portugal	Behavioral disorders and hallucinations	46
Lv et al. [10]	2023	43	Male	China	Five-year chronicle of delusions, hallucinations, and irritability	46

HD is a rare autosomal dominant neurological disorder characterized by complete penetrance [3,5]. It typically manifests between the ages of 30 and 50. The causative gene, identified in 1993, resides on the short arm of chromosome 4, specifically within its N-terminal segment. Disease onset is triggered by an expansion of CAG trinucleotide repeats within the huntingtin gene. A genetic diagnosis confirms HD when the number of CAG triplets exceeds 36. The phenotypic expression of the disease correlates with alleles surpassing 35 repeats, while those below 27 repeats are generally non-pathogenic. Research suggests a correlation between a higher number of CAG repeats and an earlier disease onset, with juvenile forms emerging before age 20 when repeats exceed 55 [3,5]. Our case of the disease manifesting at the age of 29 with 47 triplets is consistent with the typical range of its onset. 

Anatomically, HD is characterized by bilateral striatal atrophy, impacting the caudate nucleus and putamen, with up to 90% neuronal loss in juvenile cases. Additional affected areas include the globus pallidus, subthalamic nucleus, and substantia nigra. Atrophy extends to the cerebral cortex, cerebellum, hippocampus, hypothalamus, and thalamus, precipitating progressive neurodegeneration and neuronal death [11].

Initially, HD typically presents with psychiatric, neurological, and cognitive symptoms. While two-thirds of cases exhibit solely physical symptoms like chorea, dysarthria, and dystonia, the remaining third manifests psychiatric symptoms alone [3,12]. Our patient's presentation solely with psychiatric symptoms at onset aligns with this pattern. Among the psychiatric manifestations of HD, affective disorders such as depression and anxiety are common, with prevalence rates ranging from 35% to 41% across different studies [3,5,8,13]. Psychotic symptoms and hallucinations, less common yet present in 6% to 25% and approximately 1% to 3% of cases respectively [12,14], often co-occur with motor symptoms or escalate in advanced stages [3,14]. Our case is noteworthy for its initial presentation mirroring schizophreniform disorder, with negative symptoms and disorganization, which initially led to a misdiagnosis. Excessive alcohol consumption may have exacerbated behavioral disorders and contributed to Wernicke's encephalopathy, which clinically resembled HD and complicated the diagnosis. While psychotic manifestations with hallucinatory activity typically escalate as HD progresses, they are rare in its early stages, regardless of neurological symptoms [14].

Symptoms such as apathy or abulia, commonly attributed to HD, can also mimic the negative symptomatology of psychotic disorders, making them very difficult to distinguish. Cognitive symptoms in HD typically manifest as simple memory loss, difficulties in learning and retaining new information, or recall disorders progressing to dementia [3]. The present case exhibited cognitive symptoms, evidenced by a MMSE score of 22/30, likely exacerbated by regular alcohol consumption.

Offspring of the affected families by HD have an elevated risk of mental and personality disorders, possibly due to familial stressors contributing to the induction of affective disorders and emotional instability [15]. In the case of our patient, who left her family environment for a foster one, these familiar stressors did not seem relevant. An alternative hypothesis suggests that the reduction of dopamine receptors in the striatum may be a contributing factor. However, not all individuals with reduced receptors develop psychotic symptoms [16]. Moreover, the early atrophy of the caudate nucleus is employed to elucidate the psychotic alterations that are observed in individuals with HD [17], necessitating further investigation into early caudate nucleus atrophy's role in psychotic changes observed in HD. In fact, no correlation has been established between CAG triplet expansions and HD's psychiatric manifestations [3,18].

This observation illustrated the diagnostic challenges posed by complex clinical contexts and initial comorbidities, including alcohol dependence, social vulnerability, and alcohol-induced neuropsychiatric complications such as alcohol withdrawal syndrome, delirium tremens, and alcohol-induced psychotic disorder. Initially misdiagnosed with schizophreniform psychosis in the absence of an organic cause, the deleterious effect of alcohol further complicated the clinical picture, potentially precipitating Wernicke's encephalopathy. While Gayet-Wernicke encephalopathy and HD have distinct pathophysiologies, they share clinical similarities, particularly in their early stages when neuromotor symptoms are subtle or absent, encompassing cognitive, oculomotor, and psychiatric manifestations [19].

One of the limitations of this paper is that additional information could have been obtained from magnetic resonance imaging and electroencephalogram tests, which were not performed due to the patient's lack of cooperation. This case is further limited as no standardized scales for assessing the severity of psychotic symptoms, such as the Positive and Negative Syndrome Scale, were applied.

## Conclusions

This case presentation elucidates the intricate clinical reasoning process amidst psychotic symptoms initially misconstrued as primary psychosis. The confluence of alcohol addiction comorbidity and the association with Wernicke's encephalopathy posed diagnostic challenges, obscuring the recognition of an organic disease. Only through meticulous exploration of the family's medical history did the diagnostic trajectory veer toward the etiology of the psychotic symptoms. It is imperative to not attribute symptoms to primary psychiatric causes but rather diligently pursue an organic diagnosis, given that treatment for psychotic symptomatology hinges on addressing the underlying etiology.
